# A novel bibliometric index with a simple geometric interpretation

**DOI:** 10.1371/journal.pone.0200098

**Published:** 2018-07-10

**Authors:** Trevor Fenner, Martyn Harris, Mark Levene, Judit Bar-Ilan

**Affiliations:** 1 Department of Computer Science and Information Systems, University of London, London WC1E 7HX, United Kingdom; 2 Department of Information Science, Bar-Ilan University, Ramat Gan, Israel; CPERI, GREECE

## Abstract

We propose the *χ*-index as a bibliometric indicator that generalises the *h*-index. While the *h*-index is determined by the maximum square that fits under the citation curve of an author when plotting the number of citations in decreasing order, the *χ*-index is determined by the maximum area rectangle that fits under the curve. The height of the maximum rectangle is the number of citations *c*_*k*_ to the *k*th most-cited publication, where *k* is the width of the rectangle. The *χ*-index is then defined as $\sqrt{kc_{k}}$, for convenience of comparison with the *h*-index and other similar indices. We present a comprehensive empirical comparison between the *χ*-index and other bibliometric indices, focusing on a comparison with the *h*-index, by analysing two datasets—a large set of Google Scholar profiles and a small set of Nobel prize winners. Our results show that, although the *χ* and *h* indices are strongly correlated, they do exhibit significant differences. In particular, we show that, for these data sets, there are a substantial number of profiles for which *χ* is significantly larger than *h*. Furthermore, restricting these profiles to the cases when *c*_*k*_ > *k* or *c*_*k*_ < *k* corresponds to, respectively, classifying researchers as either tending to *influential*, i.e. having many more than *h* citations, or tending to *prolific*, i.e. having many more than *h* publications.

## 1 Introduction

The debate in bibliometrics on quality versus quantity in evaluating academic research performance is still an ongoing concern [1]. One perspective is to view the number of publications of a researcher (*P*) as a measure of quantity and the total number of citations to these publications (*C*) as a perceived measure of quality; several variants of these, such as the average number of citations per publication, the number of citations to the top or the 10th most cited publication, and the number of publications with at least 10 citations, have also been suggested [2]. Although these simple metrics tend to take into account only one facet of a researcher’s impact, several other bibliometric indices, such as the *h*-index [3], the *g*-index [4] and generalisations of these [5], combine both citation and publication counts.

An extensive review of the *h*-index and some of its variants was provided by Egghe in [6], and, a comparison of 37 variants of the *h*-index was given by Bornmann et al. in [7]. In addition, Waltman and van Eck [8] discussed a number of inconsistencies of the *h*-index and its variants, and proposed a family of bibliometric indicators that do not suffer from these inconsistency problems. Of particular interest are extensions of the *h*-index, which take into account the full publication list of a researcher such as the *tapered* *h*-index [9]. Proposals for new variants of the *h*-index continue to appear, for example [10–13], as do comparisons and evaluations, for example [14, 15].

Nevertheless, the *h*-index and its variants do not normally take into account the full citation list of a researcher. This could be perceived as a drawback; however, the total citation count has the disadvantage of biasing the index in favour of researchers with very highly-cited top publications or very many publication with a relatively small number of citations. We now review the *h*-index and some of its variants, and then introduce the *χ*-index, a new index that addresses some of the drawbacks mentioned.

The *h-index* of a researcher is the maximum number *h* of the researcher’s publications such that each has at least *h* citations [3]. Equivalently, consider the *citation vector*, 〈*c*_1_, *c*_2_, …, *c*_*n*_〉 of a researcher, where the *c*_*i*_, the number of citations to publication *i*, are sorted in descending order, i.e. *c*_*i*_ ≥ *c*_*j*_ if *i* < *j*. Here we assume that for all *i*, *c*_*i*_ > 0, and that *h* will be zero in the absence of any citations; this is consistent with defining the value of a bibliometric index of a researcher to be zero if none of the researcher’s publications have been cited [16]. The *h*-index is thus the largest rank *h* for which *c*_*h*_ ≥ *h*. The *h*-index is completely insensitive to the fact that a researcher’s top few publications may be very highly cited, and conversely also to a researcher having a fair number of publications whose number of citations is less than but close to *h* [17]. A suggested improvement over the *h*-index, which gives extra weight to highly cited publications, is the *g-index*. The *g*-index of a researcher is the largest rank *g* for which $\sum_{i=1}^{g}c_{i}\geq g^{2}$ [4]; it is easily shown that *g* ≥ *h*. A problem with the *g*-index is that it may still be biased since, if a researcher has a few publications that are very highly cited and the rest have very few citations, the *g*-index will still be high. This is because the *g*-index is equal to the largest rank *g* such that the average number of citations up until that rank is at least *g*. Suppose the *h*-index of a researcher is *h*, then the *h-core* is the set of the *h* most highly-cited publications for this researcher. The *A-index*, which is the average number of citations to the publications in the *h*-core, i.e. $A=\sum_{i=1}^{h}c_{i}/h$, was defined as an attempt to address the fact that the *h*-index does not take into account the total number of citations to publications in the *h*-core [18]. However, the *A*-index suffers from the fact that taking an average will, all other things being equal, often favour authors with fewer publications when they are highly cited. To remedy this issue, the *R-index* has been proposed, where $R=\sqrt{\sum_{i=1}^{h}c_{i}}=\sqrt{Ah}$ [18]. It is easy to see that *h* ≤ *R* ≤ *A*. Nevertheless, the *A* and *R* indices, and to a lesser extent the *g*-index, ignore the effect of publications outside the *h*-core, which are also part of a researcher’s output. A recent proposal is the *Euclidean-index* [19] (which we call the *E*-index), designed to take account of the full list of an author’s cited publications; it is defined as the Euclidean norm of the citation vector, i.e. $E=\sqrt{\sum_{i=1}^{n}c_{i}^{2}}$.

**Fig 1:**
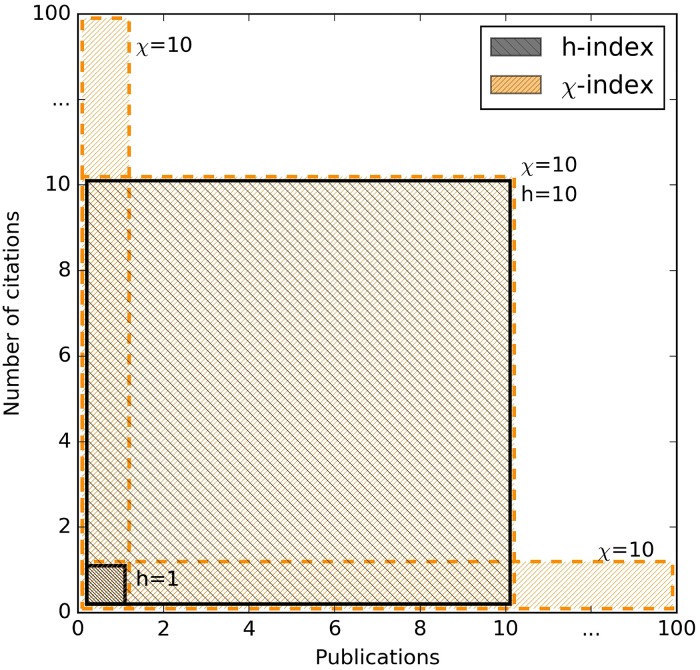
Example of the geometric interpretation of the *h* and *χ* indices.

In order to motivate the *χ*-index, we first observe that, given a citation vector for a researcher, for any *k*, *k* ≤ *n*, the researcher has at least *k* publications with *c*_*k*_ or more citations. It follows that the *h*-index is the largest *h* such that *c*_*h*+1_ ≤ *h*, i.e. for all *h*′ > *h*, *c*_*h*′_ ≤ *h*. So, for example, if one author has a single publication with 100 citations and another has 10 publications each with 10 citations, then the *h*-index of the former is 1 while the *h*-index of the latter is 10. At the other extreme, an author with 100 publications, each with a single citation, has an *h*-index of 1. The argument for favouring publications with a higher number of citations is normally that of quality versus quantity. However, such an approach, on the one hand, disadvantages a researcher with a few very highly cited publications, who may have carried out some very *influential* seminal research, whilst, on the other hand, it also disadvantages a *prolific* researcher who may have many collaborators but fewer citations per publication. Avoiding the debate of number of citations versus number of publications, we propose an index for which all three afore-mentioned scenarios, (i) 1 publication with 100 citations, (ii) 10 publications with 10 citations each, and (iii) 100 publications with 1 citation each, are considered as equally desirable. So the *χ*-index is essentially the largest product *ic*_*i*_ where 1 ≤ *i* ≤ *n*; however, for comparison purposes with the *h*-index, we will actually define the *χ*-index to be the square root of this, i.e. $\sqrt{ic_{i}}$. Thus, in all three scenarios the *χ*-index of the researcher is 10; see Fig 1, which illustrates the three scenarios in a geometrical context. If we let *k* denote the value of *i* that maximises *ic*_*i*_, we see that in all three cases, the researcher has exactly *k* publications with *c*_*k*_ or more citations. It is clear that the *h*-index cannot be larger than the *χ*-index, since $\sqrt{hc_{h}}\geq h$.

A possible future line of research would be to investigate pairwise combinations of the *χ*-index with other indices, along the lines of the two-variable metrics examined in [2].

The *χ*-index is formally introduced in Section 2, generalising the *h*-index by allowing the interplay between *k* (the number of publications, representing quantity) and *c*_*k*_ (the number of citations, representing quality). We also list some properties of the *χ*-index, which could form the basis of its axiomatisation (cf. [16, 20]), and explain the computational methods we use for the empirical analysis in the following sections. In Section 3 we introduce the two data sets analysed, a large Google Scholar data set, described in Subsection 3.1, and a small data set of Nobel prize winners, described in Subsection 3.2. In Section 4 we present the main analysis of the data sets and results obtained. In Subsection 4.1 we analyse the Google Scholar data set, and in Subsection 4.1 we turn our attention to the Nobel prize winners data set. Our main tool here is to partition the researchers into three classes, (i) when *k* is approximately equal to *h*, (ii) when *k* is significantly greater than *h* and (iii) when *k* is significantly less than *h*. We further partition that data according to whether *χ* is approximately equal to *h* or significantly larger than *h* to get a sense of when these two indices differ. Membership of the classes is determined by a *basic bootstrap percentile method* [21], Section 5.3.1] described in Section 2. In Section 5 we give our concluding remarks. (We note that we use the terms author and researcher interchangeably).

## 2 Methods

The *citation curve* is the curve resulting from plotting the number of citations against the ranking of the publications, as specified by the citation vector. The *χ*-index is the square root of the maximum area rectangle that can fit under the citation curve (see Fig 1). Formally,

$$\begin{array}{c} \chi =\sqrt{\max_{i}ic_{i}}, \end{array}$$ (1)

where *c*_*i*_ is the number of citations to publication *i* in the citation vector 〈*c*_1_, *c*_2_, …, *c*_*n*_〉, which represents all cited publications in decreasing order of the number of citations. In the following we let *k* denote the value of *i* that maximises *ic*_*i*_.

We note that, since square root is monotonic, it does not affect the ranking of researchers implied by (1). It is, however, convenient for comparison with the *h*-index and its derivatives. This can be viewed as the requirement from physics, known as *dimensional homogeneity*, that we only compare quantities that have the same units [22]. The square root accords with the geometrical interpretations of the *h* and *χ* indices: the *h*-index is the square root of the area of the maximal square that fits under the citation curve [23], and the *χ*-index the square root of the area of the maximal rectangle. It could also be interesting to consider aggregate functions other than the maximum in (1), for example, minimum, average or average of the minimum and maximum, although these seem to be rather less intuitive in the context of bibliometrics.

Several researchers have studied various properties of citation indices [16, 20] in an attempt to provide objective justification for comparison between indices, and where possible to obtain an axiomatisation of the indices. We list some properties of the *χ*-index, desirable properties that the *χ*-index possesses and one that it does not; we leave a complete axiomatisation of the *χ*-index to future work.

1. $\chi \geq \sqrt{n}$ and $\chi \geq \sqrt{c_{1}}$, where *n* is the number of cited publications and *c*_1_ is the number of citations to the most highly cited publication.
2. for all *i*, $\sqrt{ic_{i}}\leq \chi \leq \sqrt{\sum_{i=1}^{n}c_{i}}$.
3. *h* ≤ *χ*.
4. The *χ*-index is *monotonic* [16, 19], in the sense that adding citations to an existing publication or adding a new publication to the list do not lower the index. (Note that the *h*-index is also monotonic).
5. The *χ*-index is *scale-invariant* [19], in the sense that multiplying the number of citations to each publication by a constant does not change the relative ranking of two citation vectors. (Note that the *h*-index is not scale-invariant).
6. The *χ*-index is not *independent* [19], since adding a new paper with the same number of citations to two citation vectors may change their relative ranking. For example, the *χ* indices of both 〈2, 2〉 and 〈1, 1, 1, 1〉 are 2, however the *χ*-index of 〈2, 2, 1〉 is still 2 but the *χ*-index of 〈1, 1, 1, 1, 1〉 is $\sqrt{5}$. (Note that the *h*-index is also not independent).

In the following sections we carry out an empirical analysis of the *χ*-index, comparing it to the citation indices mentioned in the introduction, however, focusing our attention on the comparison of the *χ*-index and the *h*-index. We make use of a large data set compiled by Radicchi and Castellano from Google Scholar [24], and also analyse a small data set of 99 Nobel prize winners; both are described in Section 3.

Our initial comparison between the indices is carried out using the Spearman rank-correlation coefficient [25], which demonstrates that the indices we are comparing are all highly correlated, except for *P*, the number of cited publications. We carry out a more in-depth comparison of the *χ* and *h* indices in Section 4, by separating authors whose *χ* and *h* indices are approximately the same from those for which they are significantly different.

We make use of the *bootstrap method* [21], which is a technique for computing a statistic that relies on random resampling with replacement from a given sample data set. The bootstrap method is usually nonparametric, making no distributional assumptions about the data set employed. In its basic form, for example, it can be used to estimate the distribution of the population mean by computing sample means over a large number of bootstrap resamples taken from the original data set. The specific method we use to classify the authors is the *basic bootstrap percentile method* [21], Section 5.3.1]; see also [26], which also uses the bootstrap method in the context of bibliometrics. In particular, we resample author citation vectors 1000 times, with replacement, compute the *h*-index for each resample, and then compute a 99% one-sided confidence interval for the *h*-index values, starting from the lowest one from the 1000 resamples. This allows us to determine for a given author whether *k* is approximately equal to *h* and, additionally, whether *χ* is approximately equal to *h* by checking whether *k* or *χ* are in the confidence interval or not.

We thus first partition the authors into three classes, according to whether (i) *k* ≈ *h*, (ii) *k* > *h*, or (iii) *k* < *h*, where ≈ means approximately equals. The second and third classes capture a tendency of an author towards being *prolific* when *k* > *h*, or *influential* when *k* < *h*. (This does not imply that when *k* ≈ *h* the researcher is not prolific or influential, rather the distinction is meant to highlight the two opposing cases). We further partition each class according to whether *χ* ≈ *h* or *χ* > *h* to see when the indices differ, and to get a sense of the proportion of researchers for which *χ* ≈ *h*. Finally, we also consider the subclasses of *χ* > *h*, depending on whether *c*_*k*_ > *k* or *c*_*k*_ < *k*.

## 3 Data sets and preliminary analysis

We now introduce the two data sets, provide some basic statistics of these data sets, and compute the correlations between various indices for the researchers concerned. In Subsection 3.1 we consider the Google Scholar data set and in Subsection 3.2 we consider a data set of Nobel prize winners.

### 3.1 Google Scholar data set

For our main analysis, we made use of a large data set of Google Scholar profiles compiled and made available by Radicchi and Castellano [24]. The full data set contains approximately 90,000 citation vectors of authors across all disciplines, collected between June 29 and July 4, 2012. As in [24], we only included authors who had validated their Google Scholar account, and we removed authors with fewer than twenty publications, publications with no citations and publications dated before 1945. We then filtered the data further to include only authors having a career of five years or more, where the career is deemed to begin from the year of the first published paper within the window of years considered. After this preprocessing step, the final data set we used was reduced to 34,393 citation profiles.

We start by presenting, in Table 1, the basic statistics for the various indices introduced in Section 1; *h*, *g*, *A*, *R*, $\sqrt{E}$, *χ*, $\sqrt{C}$ and *P*, stand for the *h*-index, the *g*-index, the *A*-index, the *R*-index, the square root of the Euclidian-index, the *χ*-index, the square root of the total number of citations and the number of publications, respectively. (We note that we have chosen to use $\sqrt{E}$ and $\sqrt{C}$ for comparison purposes). It can be seen that the number of cited publications *P* stands out as a clear outlier, and also *A*, to a lesser extent. Moreover, apart from **min**, the statistics for *h* are the lowest, closely followed by $\sqrt{E}$.

**Table 1:** Basic statistics for various indices for the Google Scholar data set.

	*h*	*g*	*A*	*R*	$\sqrt{E}$	*χ*	$\sqrt{C}$	*P*
**mean**	18.85	35.12	68.22	34.20	19.39	23.57	39.05	68.60
**median**	15.00	27.00	18.00	27.00	15.64	19.05	30.76	46.00
**min**	2.00	3.00	1.00	3.00	3.11	4.58	6.33	20.00
**max**	213.00	366.00	1648.00	333.00	219.39	220.69	396.30	3684.00
**std**	12.44	25.98	35.35	26.14	13.80	16.09	28.26	70.41

In Table 2, we present the Spearman rank-correlation coefficient **r** [25] between the various indices, noting that when computing the Pearson correlation [25] the results were similar; due to symmetry we only present the upper triangle of the correlation matrix. (We note that while the Pearson correlation measures the strength of a linear association between two random variables, the Spearman rank-correlation measures the strength of a monotonic association between the two, which may be nonlinear [27]). We observe that *P* has the lowest correlation with any of the other indices, and that all the other indices are highly correlated with each other. We further note that, although $\sqrt{C}$ is indeed highly correlated with all the other indices apart from *P*, it has a possible perceived disadvantage, as do *P* and $\sqrt{E}$, in that it takes into account the complete list of publications.

**Table 2:** Spearman rank-correlation between the various indices computed from the Google Scholar data set.

Spearman **r**	*h*	*g*	*A*	*R*	$\sqrt{E}$	*χ*	$\sqrt{C}$	*P*
*h*	1.000	0.955	0.816	0.931	0.860	0.932	0.952	0.808
*g*		1.000	0.918	0.974	0.943	0.963	0.977	0.762
*A*			1.000	0.969	0.991	0.947	0.938	0.532
*R*				1.000	0.982	0.988	0.990	0.672
$\sqrt{E}$					1.000	0.964	0.962	0.600
*χ*						1.000	0.990	0.698
$\sqrt{C}$							1.000	0.754
*P*								1.000

From now on, we will concentrate on comparing the *h* and *χ* indices, *h* being the most commonly employed index, and leave detailed comparison to other indices for future work.

We start by showing, as was done in [24], that the probability density functions of the *h* and *χ* indices both follow log-normal distributions [28, 29]. To this end we introduce the *Jensen-Shannon divergence* (*JSD*) [30], which is a nonparametric measure of the distance between two empirical distributions **p** = (*p*_*i*_) and **q** = (*q*_*i*_), where *i* = 1, 2, …, *n*.

The formal definition of the *JSD*, which is a symmetric version of the Kullback-Leibler divergence and is based on Shannon’s entropy [31], is given by

$$\begin{array}{c} JSD\left(p,q\right)=\sqrt{\frac{1}{2\ln2}\;\sum_{i=1}^{n}(p_{i}\;\ln\frac{2p_{i}}{p_{i}+q_{i}}+q_{i}\;\ln\frac{2q_{i}}{p_{i}+q_{i}})}, \end{array}$$ (2)

where we use the convention that if *p*_*i*_ = 0 or *q*_*i*_ = 0, or both, 0 ln 0 and 0 ln (0/0) are both defined to be 0. (The factor 2 ln 2 is included to normalise the *JSD* to be between 0 and 1). We observe that the JSD is equal to 0 when **p** = **q**.

In Table 3 we give the mean *μ*, and standard deviation *σ* of the log-normal distributions fitted by the maximum likelihood method, and the JSD between the empirical distributions of the *h* and *χ* indices and the fitted log-normal distributions. The low JSD values indicate good fits for both indices. We also note that the means and standard deviations are quite close.

**Table 3:** Maximum likelihood fitting of log-normal distributions to the *h* and *χ* indices of the Google Scholar data set.

Parameter	*μ*	*σ*	*JSD*
*h*-index	2.768	0.565	0.012
*χ*-index	2.985	0.575	0.009

### 3.2 Nobel prize winners data set

For our second data set, we collected the citation vectors of 99 Nobel prize winners across a variety of disciplines from the Web of Science platform [32]. We included only authors having twenty or more publications, and only those publications with citations. However, for this data set we considered their full careers without a cutoff date. In Table 4, we present the basic statistics for the Nobel laureates, while in Table 5 we present the Spearman rank-correlation coefficient. As one would expect, the statistics are, overall, much higher than for the Google Scholar data set, although for this data set *A* is more of an outlier than *P*. On the other hand, the correlations are comparable to the Google Scholar data set, although, on average lower.

**Table 4:** Basic statistics for various indices for the Nobel prize winners data set.

	*h*	*g*	*A*	*R*	$\sqrt{E}$	*χ*	$\sqrt{C}$	*P*
**mean**	66.60	136.02	320.89	140.06	67.06	86.13	153.24	214.54
**median**	65.00	128.00	289.00	136.00	61.38	84.29	148.94	165.00
**min**	12.00	20.00	53.00	25.00	16.20	17.55	26.02	20.00
**max**	195.00	319.00	1452.00	379.00	202.82	213.86	384.62	1139.00
**std**	35.47	69.16	210.20	66.20	31.71	39.09	73.01	178.28

**Table 5:** Spearman rank-correlation between the various indices computed from the Nobel prize winners data set.

Spearman **r**	*h*	*g*	*A*	*R*	$\sqrt{E}$	*χ*	$\sqrt{C}$	*P*
*h*	1.000	0.941	0.524	0.891	0.616	0.879	0.930	0.884
*g*		1.000	0.657	0.934	0.732	0.884	0.941	0.887
*A*			1.000	0.844	0.960	0.796	0.759	0.389
*R*				1.000	0.852	0.939	0.968	0.739
$\sqrt{E}$					1.000	0.834	0.820	0.487
*χ*						1.000	0.969	0.733
$\sqrt{C}$							1.000	0.812
*P*								1.000

In Table 6 we show the parameters of the log-normal distribution fitted by the maximum likelihood method, and the JSD between the empirical distributions of the *h* and *χ* indices and the fitted log-normal distributions. As for the Google Scholar data set, the low JSD values indicate good fits for both indices. We again note that the means and standard deviations are quite close.

**Table 6:** Maximum likelihood fitting of log-normal distributions to the *h* and *χ* indices of the Nobel prize winners data set.

Parameter	*μ*	*σ*	*JSD*
*h*-index	4.048	0.576	0.034
*χ*-index	4.352	0.472	0.029

## 4 Analysis and results

We now analyse the data sets introduced in Section 3, with the aim of revealing how authors are separated into classes depending on whether *k* ≈ *h* or not, or whether *χ* ≈ *h* or not. In Subsection 4.1 we analyse the Google Scholar data set, and in Subsection 4.2 we analyse the Nobel prize winners data set.

### 4.1 Results for Google Scholar data set

**Fig 2:**
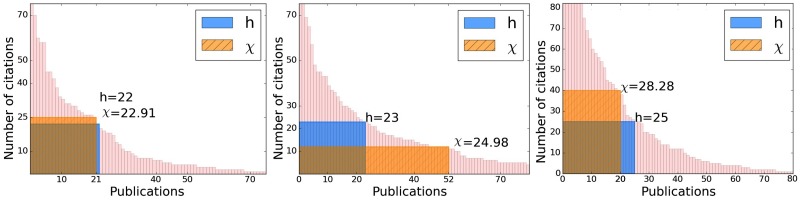
Examples of authors for the Google Scholar data set: *k* ≈ *h* (left) *k* > *h* (middle) *k* < *h* (right).

In Fig 2, we see three examples of authors according whether (i) *k* ≈ *h*, (ii) *k* > *h*, or (iii) *k* < *h*, exhibiting the geometry of the *h* and *χ* indices. When *k* > *h* there are many publications, each with fewer than *h* citations (tending towards *prolific*), and when *k* < *h* therefore fewer publications, each with more than *h* citations (tending towards *influential*).

In Table 7, we exhibit the breakdown of the three classes for the Google Scholar data set, noting that *k* < *h* is the largest class, the other two comprising just over 53.50% of the data set. It is also apparent that, within the class *k* < *h*, there are by some margin, more authors for which *χ* > *h*. What this means is that, when *χ* is significantly larger than *h*, we expect that *k* will be significantly smaller than *h*, i.e. we expect the author to have several publications with more than *h* citations, contributing to *χ* being larger *h*; this can be justified from the data in Table 7 with the use of Bayes theorem. This confirms that the *χ*-index addresses a problem of the *h*-index that it does not sufficiently take into account highly cited publications. The statistics in Table 8 for the three classes further confirm this property of the *χ*-index, showing higher average values for the *χ*-index when *k* < *h*.

**Table 7:** Breakdown of the three *k* classes for the Google Scholar data set.

Class	# authors	% authors	% *χ* ≈ *h*	% *χ* > *h*
*k* ≈ *h*	11764	34.20%	93.21%	6.79%
*k* > *h*	6675	19.41%	88.97%	11.03%
*k* < *h*	15954	46.39%	47.01%	52.99%

**Table 8:** Basic statistics for *k* ≈ *h* (left) *k* > *h* (centre) *k* < *h* (right) for the Google Scholar data set.

*k* ≈ *h*	*χ*	*h*	*k* > *h*	*χ*	*h*	*k* < *h*	*χ*	*h*
**mean**	18.73	16.85	**mean**	19.92	18.02	**mean**	28.67	20.68
**median**	15.49	14.00	**median**	15.49	14.00	**median**	23.75	17.00
**min**	4.58	2.00	**min**	4.58	3.00	**min**	5.00	3.00
**max**	140.43	139.00	**max**	165.96	159.00	**max**	220.69	213.00
**std**	11.06	10.80	**std**	14.14	13.53	**std**	18.31	12.83

Moreover, it can be seen in Table 9 that out of all authors, there are 28.60% for which *χ* is significantly larger than *h*, clearly demonstrating the potential of the *χ*-index to separate authors that may have similar *h* indices. In addition, the statistics shown in Table 10 indicate higher average values when *χ* > *h*. The breakdown of the *χ* > *h* class, when *c*_*k*_ > *k* and *c*_*k*_ < *k*, can be seen in Table 11, while the basic statistics pertaining to these classes are shown in Table 12. It can be seen that the average values for the larger subclass, *c*_*k*_ > *k*, are much higher than those for the smaller subclass, *c*_*k*_ < *k*.

**Table 9:** Breakdown of the two *χ* classes for the Google Scholar data set.

Class	# authors	% authors
*χ* ≈ *h*	24558	71.40%
*χ* > *h*	9835	28.60%

**Table 10:** Basic statistics for *χ* ≈ *h* (left) and *χ* > *h* (right) for the Google Scholar data set.

*χ* ≈ *h*	*χ*	*h*	*χ* > *h*	*χ*	*h*
**mean**	20.59	18.56	**mean**	31.00	19.59
**median**	16.70	15.00	**median**	25.55	16.00
**min**	4.58	3.00	**min**	4.69	2.00
**max**	214.90	213.00	**max**	220.69	106.00
**std**	13.10	12.53	**std**	19.98	12.19

**Table 11:** Further breakdown of the *χ* > *h* class for the Google Scholar data set.

Class	# authors	% authors
*c*_*k*_ > *k*	9141	92.94%
*c*_*k*_ < *k*	694	7.06%

**Table 12:** Basic statistics for the *χ* > *h* class, when *c*_*k*_ > *k* (left) and *c*_*k*_ < *k* (right) for the Google Scholar data set.

*c*_*k*_ > *k*	*χ*	*h*	*c*_*k*_ < *k*	*χ*	*h*
**mean**	31.94	19.93	**mean**	18.69	15.15
**median**	26.27	16.00	**median**	13.42	10.00
**min**	5.00	2.00	**min**	4.69	3.00
**max**	220.69	105.00	**max**	130.12	106.00
**std**	19.98	12.04	**std**	15.38	13.27

### 4.2 Results for Nobel prize winners data set

**Fig 3:**
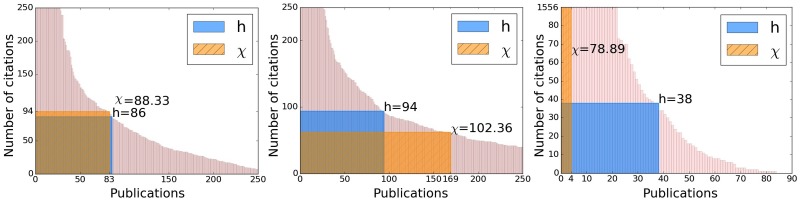
Examples of authors for the Nobel prize winners data set: *k* ≈ *h* (left) *k* > *h* (middle) *k* < *h* (right).

The Nobel prize winners data set looks at the extreme case of researchers having, on average, very high *h* values and therefore also very high *χ* values. In Fig 3 we see three examples of authors according to the three classes as in Fig 2, exhibiting the geometry of these classes for the *χ*-index for this data set. These examples can be contrasted to the ones shown in Fig 2 for the Google Scholar data set, demonstrating more extreme cases of the *χ*-index when *k* > *h* or *k* < *h*.

In Table 13, we see a significant difference from the Google Scholar data set, since for about 80% of the laureates we have *k* < *h* and, of those, for over 75% of the authors *χ* > *h*. As expected, this implies that, overall, Nobel prize winners are *influential*. Looking at the statistics in Table 14, we see that when *k* < *h*, on average, the *χ* values of researchers are much larger than the *h* values. This is due to publications with a large number citations, significantly more than *h*. An interesting observation is that unlike Table 8, where the values of the *χ*-index are the highest when *k* < *h*, in Table 14 *χ* is highest for the smaller class when *k* > *h*. This is most likely due to a long tail of highly cited publications for these few laureates.

**Table 13:** Breakdown of the three *k* classes for the Nobel prize winners data set.

Class	# authors	% authors	% *χ* ≈ *h*	% *χ* > *h*
*k* ≈ *h*	5	5.05%	100.00	0.00
*k* > *h*	15	15.15%	80.00	20.00
*k* < *h*	79	79.80%	25.32	75.64

**Table 14:** Basic statistics for *k* ≈ *h* (left) *k* > *h* (centre) *k* < *h* (right) for the Nobel prize winners data set.

*k* ≈ *h*	*χ*	*h*	*k* > *h*	*χ*	*h*	*k* < *h*	*χ*	*h*
**mean**	64.95	63.40	**mean**	110.62	103.87	**mean**	82.60	59.22
**median**	65.51	65.00	**median**	109.40	105.00	**median**	78.85	52.50
**min**	42.00	41.00	**min**	31.18	31.00	**min**	17.55	12.00
**max**	88.33	86.00	**max**	204.12	195.00	**max**	213.86	155.00
**std**	18.63	18.58	**std**	43.76	42.78	**std**	37.64	30.14

In contrast to Table 9, it can be seen from Table 15 that *χ* > *h* for over 60% of laureates. However, as the statistics in Table 16 reveal, in contrast to Table 10, the *h*-index for those Nobel prize winners with *χ* ≈ *h*, is actually, on average, higher than both the *h* and *χ* indices of the laureates with *χ* > *h*. This may indicate that for very *influential* researchers, such as Nobel laureates, when *χ* > *h* the *h*-index undervalues their contribution. The breakdown of the *χ* > *h* class, when *c*_*k*_ > *k* and *c*_*k*_ < *k*, can be seen in Table 17, while the basic statistics pertaining to these classes are shown in Table 18. It is interesting to note that as opposed to the Google scholar statistics shown in Table 12, the average values for the Nobel laureates subclass *c*_*k*_ > *k* are, in fact, much lower than those for the subclass *c*_*k*_ < *k*. This latter class is quite small as there are only three such Nobel prize winners; see Table 18. As noted above this is most likely due to a long tail of relatively highly cited publications for these few laureates.

**Table 15:** Breakdown of the two *χ* classes for the Nobel prize winners data set.

Class	# authors	% authors
*χ* ≈ *h*	37	37.37%
*χ* > *h*	62	62.63%

**Table 16:** Basic statistics for *χ* ≈ *h* (left) and *χ* > *h* (right) for the Nobel prize winners data set.

*χ* ≈ *h*	*χ*	*h*	*χ* > *h*	*χ*	*h*
**mean**	92.69	88.00	**mean**	82.22	53.84
**median**	91.39	86.00	**median**	73.67	49.50
**min**	29.73	24.00	**min**	17.55	12.00
**max**	204.12	195.00	**max**	213.86	136.00
**std**	38.85	37.97	**std**	39.01	26.93

**Table 17:** Further breakdown of the *χ* > *h* class for the Nobel prize winners data set.

Class	# authors	% authors
*c*_*k*_ > *k*	59	95.16%
*c*_*k*_ < *k*	3	4.84%

**Table 18:** Nobel prize winners basic statistics for the *χ* > *h* class, when *c*_*k*_ > *k* (left) and *c*_*k*_ < *k* (right).

*c*_*k*_ > *k*	*χ*	*h*	*c*_*k*_ < *k*	*χ*	*h*
**mean**	81.20	52.10	**mean**	102.40	88.00
**median**	70.40	48.00	**median**	100.16	78.00
**min**	17.55	12.00	**min**	85.46	78.00
**max**	213.86	136.00	**max**	121.59	108.00
**std**	39.59	26.25	**std**	18.17	17.32

## 5 Concluding remarks

We have presented a new citation index, the *χ*-index, which addresses some shortcomings of the *h*-index in terms of the balance between number of citations and number of publications. The *χ*-index has a simple geometric characterisation in terms of the largest area rectangle that fits under the citation curve; this generalises the *h*-index for which the rectangle is constrained to be a square.

We have analysed two data sets, a large one from Google Scholar and a small one of Nobel prize winners. Studying these data sets clearly shows the utility of the *χ*-index. First, as with many of the citation indices that combine number of citations (proxy for quality) with number of publications (quantity), the *χ*-index correlates strongly with the square root of the total number of citations, yet it is selective in its choice of publications to include in the index. Second, as we have seen from our analysis, there are many researchers whose *χ*-index is significantly larger than their *h*-index due to their tendency to be *influential*, in the case *k* < *h*, or *prolific* in the case *k* > *h*. We believe that this property of the *χ*-index is beneficial and could lead to a more satisfactory ranking of researchers than that obtained using the *h*-index.
